# The Effects of Cholera Toxin on Cellular Energy Metabolism

**DOI:** 10.3390/toxins2040632

**Published:** 2010-04-08

**Authors:** Rachel M. Snider, Jennifer R. McKenzie, Lewis Kraft, Eugene Kozlov, John P. Wikswo, David E. Cliffel

**Affiliations:** 1 Department of Chemistry, Vanderbilt University, VU Station B. Nashville, TN 37235-1822, USA; Email: rachel.m.snider@gmail.com (R.S.); jennifer.mckenzie@vanderbilt.edu (J.M.); lewis.kraft@vanderbilt.edu (L.K.); evkoz2003@mail.ru (E.K.); 2 Vanderbilt Institute for Integrative Biosystems Research and Education, Vanderbilt University, Nashville, TN 37235-1809, USA; Email: john.p.wikswo@vanderbilt.edu (J.W.); 3 Departments of Physics, Biomedical Engineering, and Molecular Physiology and Biophysics, Vanderbilt University, Nashville, TN 37235-1809, USA

**Keywords:** cholera toxin, metabolism, multianalyte, microphysiometry, cyclic AMP, forskolin, PC-12

## Abstract

Multianalyte microphysiometry, a real-time instrument for simultaneous measurement of metabolic analytes in a microfluidic environment, was used to explore the effects of cholera toxin (CTx). Upon exposure of CTx to PC-12 cells, anaerobic respiration was triggered, measured as increases in acid and lactate production and a decrease in the oxygen uptake. We believe the responses observed are due to a CTx-induced activation of adenylate cyclase, increasing cAMP production and resulting in a switch to anaerobic respiration. Inhibitors (H-89, brefeldin A) and stimulators (forskolin) of cAMP were employed to modulate the CTx-induced cAMP responses. The results of this study show the utility of multianalyte microphysiometry to quantitatively determine the dynamic metabolic effects of toxins and affected pathways.

## 1. Introduction

Cholera toxin (CTx), secreted by the gram negative bacterium *Vibrio cholera*, causes the symptoms observed in cholera, a potentially life-threatening infectious disease in the developing world [1,2]. The seventh cholera pandemic that continues today began in the 1960s when the El Tor strain of cholera emerged as the most prevalent form of the disease [1,2,3]. This strain entered the Western hemisphere in 1991 in Peru and has since caused more than a million cases in Central and South America [2]. The animal reservoirs of *V. cholerae* are shellfish and plankton, and infection can be caused by consuming contaminated water or food [1,2]. Those vibrios that survive passage through the stomach can adhere to the mucosal surface of microvilli of the small intestine where they secrete CTx [1,2] as well as other toxins [3]. The concentration of vibrios on the mucosal surface rapidly increases from 10^7^ to 10^8^ cells per gram of tissue [1]. After incubation that can vary from 6 hours to 5 days, the onset of symptoms is abrupt and is characterized by severe diarrhea that can reach a rate of 1 L/hr. This fluid contains large amounts of sodium, chloride, bicarbonate, and potassium, as well as mucus; the loss of these electrolytes causes blood volume depletion, low blood pressure, and shock [1,2,4]. The fluid loss can be so great that without proper rehydration, death can result within hours of onset [1]. 

These clinical effects arise from the action of CTx on both the epithelial and nervous cells of the intestine. CTx is an 84 kDa hexameric protein (AB_5_) consisting of a catalytic A subunit (A1 and A2 chains) and a pentameric B subunit. Each B subunit has a binding site specific for a single ganglioside GM1 glycolipid with a total capacity of five for each CTx protein complex. CTxB binds tightly to GM1 receptors at the cell surface, and the glycolipid is believed to direct the toxin to its intracellular destination. The catalytic A subunit must gain access to the cell cytosol for CTx to exert its toxic effects; therefore, after endocytosis, the CTx-GM1 complex is trafficked retrograde from the plasma membrane to early endosomes, the Golgi, and finally to the endoplasmic reticulum (ER) where the A1 chain of the toxin is able to utilize the ER-associated degradation pathway to enter the cytosol [5]. 

The mechanism of action of CTx to activate cAMP production, once in the cytosol, is well known. The A subunit of the toxin activates the heterotrimeric G-protein, GSα, *via* ADP-ribosylation. The modified Gsα loses its GTPase activity, but remains constitutively active in its GTP-bound state, causing a continuous stimulation of adenylate cyclase. Continuous adenylate cyclase activation results in excessive production of cyclic adenosine monophosphate (cAMP) [6]. The result is the secretion of chloride ions from the cell which can be measured using electrophysiology. Time-course studies using this methodology indicate CTx takes approximately 30 minutes to increase intracellular cAMP and induce chloride secretion in a T84 intestinal epithelial cell line [7].

CTx is a particularly useful subject for our study because the clinical effects and the mechanism of adenylate cyclase activation have been well described [1,2,3,4,8,9], but there is less information known about the acute and dynamic metabolic effects that occur as a result of CTx exposure to cells. These dynamic metabolic effects can be studied with the multianalyte microphysiometer (MAMP), developed in our laboratory through modifications to a commercial Cytosensor microphysiometer allowing simultaneous measurement of extracellular glucose, lactate, oxygen, and acidification [6,10,11,12]. In the MAMP, approximately 10^5^ cells are seeded between two membranes in a 3 μL chamber and perfused with media. The flow of media is periodically stopped to allow measurable consumption of glucose, oxygen, and accumulation of lactate and acid. This method allows us to calculate metabolic rates in mol∙cell^-1^∙sec^-1^ for each stop-flow period, which is every 2 minutes. The MAMP has been used to study the metabolic effects of protein toxins [6], the metabolism of cancer cells [13], and isolated murine islets (with the addition of a sensor for insulin) [14]. 

An initial MAMP study of toxin effects on metabolism showed different metabolic processes were triggered in cell lines in response to botulinum neurotoxin A, ricin, and CTx [6]. After preliminary acidification studies using a range of cell types (fibroblast, ovary, and hepatocyte), in tandem with literature that suggested the diarrheal response to CTx has a significant neurological component (up to 50%) [8], neuronal-like cells were chosen for more in depth study in the MAMP. Specifically, PC-12 pheochromacytoma cells, will serve as a useful neuronal model for our studies [15], possessing GM1 ganglioside receptors capable of binding CTx [16], and a demonstrated increase in cAMP production in response to forskolin, a direct activator of adenylate cyclase [17]. 

We hypothesized that the metabolic responses observed in our initial studies were due to increases in intracellular cAMP concentrations caused by continuous stimulation of adenylate cyclase [6]. To investigate the proposed mechanism behind the metabolic responses to CTx, two inhibitors, H-89 and brefeldin A, and the cholera toxin B subunit (CTxB) were used. CTxB is structurally similar to CTx, retaining the ability to bind to ganglioside GM1 receptors and retrograde traffic back to the ER, but fails to induce toxicity without the catalytic A subunit. Brefeldin A reversibly disrupts vesicular transport in eukaryotic cell systems, thus preventing retrograde trafficking of the toxin to the ER and ultimately delivery of CTx A1 to the cytosol in epithelial and neuronal cell lines [18,19]. H-89, a cAMP-dependent protein kinase inhibitor, has been shown to inhibit cAMP-activated protein kinase A (PKA), which is necessary for the metabolic response after CTx exposure on PC-12 cells [17]. 

In order to investigate the mechanism of the neuronal metabolic response to CTx, we sought to compare the metabolic responses due to CTx and to another activator of adenylate cyclase, forskolin. CTx [1] and forskolin [17] are both known to cause an increase in cAMP production resulting from adenylate cyclase activation, and are often used interchangeably in pharmacology and toxicology studies. CTx has been shown to potentiate the effects of forskolin [20] and *vice versa* [21]. However, while CTx and forskolin were shown to have similar effects on ileal mucosa (non-neural) cells [22], they were previously shown to have differential effects on endocrine tissue in the pituitary [23]. This is not surprising, as forskolin and CTx have different mechanisms for increasing cAMP–forskolin directly and reversibly activates adenylate cyclase, and CTx indirectly activates adenylate cyclase, *via* ADP-ribosylatation of GSα [24]. We hypothesized a similar difference in metabolic responses would be observable by investigation with our MAMP.

Herein, we demonstrate the ability of our custom-built MAMP to measure the multianalyte metabolic response of PC-12 cells to CTx using various concentrations, exposure duration, and repeated exposures. We also demonstrate the utility of MAMP to investigate the underlying mechanisms for observed cellular metabolic responses to toxins with the inhibitors, H-89 and brefeldin A. Additionally, we demonstrate significant differences in the metabolic pathways activated by forskolin compared to cholera toxin despite their similarities in adenylate cyclase activation and cAMP production.

## 2. Materials and Methods

### 2.1. Chemicals and Instrumentation

All materials were used as obtained unless otherwise noted. CTx and the CTxB subunit from *Vibrio cholerae*, forskolin from *Coleus forskohlii*, H-89 dihydrochloride hydrate, glucose oxidase (GOx, Type IIS from *Aspergillus niger*), bovine serum albumin (BSA, fraction V, 96%), glutaraldehyde (glutaric dialdehyde, 25 wt% solution in water), and oxamate were purchased from Sigma. Stabilized lactate oxidase (LOx) was purchased from Applied Enzyme Technology (Pontypool, UK). Nafion (perfluorosulfonic acid-PTFE copolymer, 5% w/w solution in ethanol) and platinum wire were purchased from Alfa Aesar. Sterile glucose solution and (L)-lactic acid were purchased from Fischer Scientific. Lyophilized alamethicin was obtained from A.G. Scientific, Inc. (San Diego, CA). Silver epoxy was purchased from Epoxy Technologies, Inc (Billerica, MA), and Loctite^®^ Hysol^®^ 1C™ structural epoxy was purchased from Henkel Technologies (Dusseldorf, Germany). Custom RPMI 1640 media modified to be 1 mM in phosphate, bicarbonate-free, and glucose-free was purchased from Mediatech (Herndon, VA). All MAMP consumables including cell inserts, spacers, and membranes were obtained from Molecular Devices Corp. (Sunnyvale, CA). 

The modified sensor head was prepared by adding four platinum electrodes to the sensor head designed by Molecular Devices, one of which acts as the secondary counter electrode. Four 0.6 mm paths are drilled through the sensor head with the hole for the counter electrode widened on the surface to ~2 mm. The counter electrode is made by melting a 0.5 mm platinum electrode to form a 1.5 mm ball at the end. Two of the working electrodes are 0.5 mm platinum wires used for glucose and lactate measurements. The third working electrode, used to measure oxygen, is a 127 µm platinum wire, which is wrapped multiple times around a 0.5 mm platinum wire for added mechanical stability, with silver epoxy used to aid the electrical contact between the two. Each wire is embedded in the sensor head with white epoxy, ground down flush to the face of the sensor head using silicon carbide grinding paper, and polished with 1 µm diamond paste. The wires extending from the sensor head are reinforced with copper socket pins to add mechanical strength and reinforced with structural epoxy. The surface of a modified sensor head was cleaned with water and ethanol, and the electrodes cleaned electrochemically by cycling in 0.5 M sulfuric acid [34].

Acidification was measured using the LAPS sensor of the Cytosensor. Oxygen is detected through direct reduction at the 127 µm electrode at –0.45 V *vs.* Ag/AgCl (2 M KCl). Glucose and lactate are measured indirectly through oxidation of hydrogen peroxide produced through the reaction of glucose oxidase (GOx) and lactate oxidase (LOx) enzymes at +0.6 V *vs.* Ag/AgCl (2 M KCl), as shown in Figure 2. GOx and LOx films are formed by hand casting onto the electrode surface a solution of the enzyme, bovine serum albumin (BSA), and glutaraldehyde. 

### 2.2. MAMP Experiments with Pheochromacytoma Cells

Pheochromacytoma cells (~5 × 10^5^ per insert, PC-12, CRL-1721 ATCC) were seeded in Corning Costar^®^ Transwell^®^ collagen coated cell culture inserts (PTFE, 3 µm pores) and allowed to grow overnight in F12K media supplemented with 15% donor horse serum and 2.5% fetal bovine serum. Heptocellular carcinoma cells (~5 × 10^5^ per insert, HepG2, HB-8065 ATCC) were seeded in Corning Costar^®^ Transwell^®^ cell culture inserts (PTFE, 3 µm pores) and allowed to grow overnight in Eagle's Minimum Essential Medium supplemented with 10% fetal bovine serum. A four channel microphysiometer and Cytosoft^®^ program (Molecular Devices) were used to control pump cycles and temperature. The current response of the amperometric sensors was measured using a multipotentiostat developed by the Vanderbilt Institute of Integrative Biosystems Research and Education. A 120 s stop flow cycle was used with 80 s at a flow rate of 100 µL per minute and a stop period of 40 s. In all experiments modified RPMI 1640 media containing 5 mM glucose was used. CTx is sold by Sigma Aldrich as a lyophilized powder that is reconstituted as a 11.75 µM solution containing 0.05 M Tris buffer salts, pH 7.5, 0.2 M NaCl, 0.003 M NaN_3_, and 0.001 M sodium EDTA. For this reason, all experiments with CTx and controls were run with 5 mM glucose modified RPMI 1640 media supplemented to contain the appropriate concentration of these compounds.

### 2.3. Experimental Protocol

Cells were seeded into inserts and allowed to adhere overnight. Once placed in the instrument, measurement of amperometric and potentiometric signals began. The cells were allowed to equilibrate for a period to determine their rate prior to exposure. The cells were treated with toxin for a period of time, and then returned to the running media to allow a period of recovery prior to treatment with alamethicin. In all experiments the sensors were calibrated with modified RMPI media with no glucose and no lactate, with 0.05 mM lactate and 1 mM glucose, with 0.1 mM lactate and 3 mM glucose, with 0.2 mM lactate and 5 mM glucose, and in some cases with 0.3 mM lactate before being returned to the running media.

### 2.4. Data Analysis

Acidification rates were determined by the Cytosoft program as described by Owicki [25]. Amperometric signals were analyzed by comparing the stop-flow results for live and dead cells. The current response of a given stop flow period, *i_p_*, is defined as the difference between the steady state current and the current at the end of the stop flow. Data is then analyzed by subtracting the response with dead cells from the response with live cells to obtain the current response due to cellular activity, Δ*i_p_*.

The concentration in the chamber at the end of the stop flow is calculated from Δ*i_p_* using the calibration at the end of the experiment. By accounting for the microfluidic volume, the number of cells present, and the length of the stop flow period, metabolic rates can be calculated in terms of mol cell^-1^ s^-1^. Modeling efforts are underway to develop methods of analyzing the data over the entire stop flow period by modeling the stop flow period as a Cottrell step and through a mathematical model. Statistical analysis was performed by grouping stop-flow rates from each replicate chamber into basal, exposure, and various post exposure groups, then averaging the replicates. Statistical significance was determined by a two-tailed paired t-test with *p* < 0.05.

## 3. Results and Discussion

Multiple cell types, including fibroblasts, ovary, and hepatocytes, were exposed to CTx, and the extracellular acidification rates were recorded. As CTx is expected to stimulate cellular processes that increase ATP utilization, our initial goal was to identify cell lines in which CTx exposure resulted in an increase in extracellular acidification as measured by a conventional Cytosensor microphysiometer. Four immortalized cell lines were tested, including fibroblasts, hepatocytes, ovary, and neuronal cells. The acidification rate increased when A9L HD2 S.C.18, HepG2, and PC-12 cells were exposed to CTx. CHO cells showed a decrease in the extracellular acidification rate when exposed to CTx. Other studies conducted by our lab have shown that lower concentrations of CTx on fibroblasts result in the same magnitude of the metabolic response, however the lag-time until onset varies with concentration [6].

**Figure 1:**
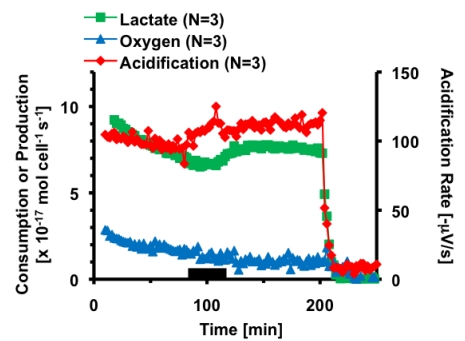
Average metabolic response of 5 × 10^5^ HepG2 cells to thirty minutes exposure to 1,000 nM CTx. The black bar indicates the 30 minutes 1,000 nM CTx was being perfused through the cell chamber. Cell necrosis is triggered at 200 minutes.

Based on these preliminary experiments, the multi-analyte metabolic response of hepatocytes to cholera was further explored. In this experiment, the basal metabolic rates of acidification, lactate production and oxygen consumption of 5 × 10^5^ HepG2 cells were measured. At eighty-two minutes, the hepatocyte cells were exposed to 1,000 nM CTx for thirty minutes, before returning control media to the cells. The cells were allowed to recover for ninety minutes before treatment with 101 µM alamethicin. Alamethicin induced cell necrosis *via* the formation of voltage-gated pores in the cell membrane, causing the metabolic rates of all signals to rapidly decrease. This cell necrosis enables the determination of the effective zero metabolic activity level for calibrating the sensors and for conversion of the raw electrochemical data into metabolic rates of mole cell^-1 ^second^-1^. Figure 1 shows the average metabolic rates of HepG2 cells as they were exposed to CTx. Three traces are shown, illustrating the calculated metabolic rates of lactate, oxygen, and acidification for each 2 minute stop-flow cycle. Oxygen is graphed as consumption, with a decrease in rate indicating a decrease in metabolic consumption of oxygen. Lactate is graphed as production, with a increase in rate indicating a increase in metabolic production. Acid production is graphed as the acidification rate on the secondary axis, or slope of the change in potential during each stop-flow cycle in -µV/sec, as provided by the Cytosensor software. Ten minutes after exposure, lactate production increased to 104 ± 4% from basal levels, however based on the rate of decrease in production prior to exposure; the actual effect on CTx on lactate production was probably larger. Acid production increased to 120 ± 8% during CTx exposure, and remained elevated until necrosis was triggered. It is important to clarify here that acidification is the sum of all acids present in the extracellular media, not only lactic acid. No significant change was seen in oxygen consumption. This metabolic change was not as significant compared to the response of fibroblasts and neuronal cells. Since we had already performed a study of CTx on fibroblasts [6], and the hepatocytes were not as responsive or relevant as the neuronal PC-12 cell line to the specific toxicological target *in vivo* of CTx, all further MAMP experiments used PC-12 cells. 

**Figure 2:**
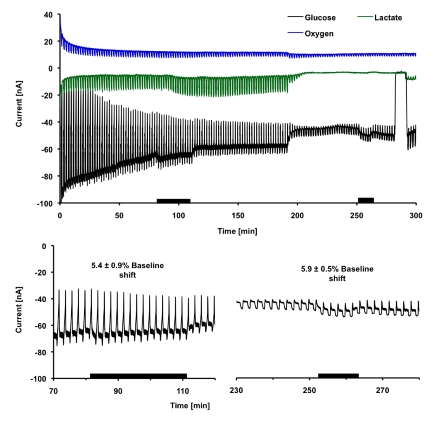
Electrochemical measurements of the metabolic response of PC-12 cells to 1000 nM Cholera Toxin. (a) Electrochemical currents corresponding to MAMP signals for 5 × 10^5^ PC-12 cells in response to 1000 nM CTx. The black bars indicate where CTx was flowed through the cell chamber. Cell death was triggered at 200 minutes, and CTx was re-introduced into the chamber to determine the effect on the sensors prior to calibration at 275 minutes. A baseline shift can be seen in the glucose signal. (b) Close-up of current at glucose electrode when CTx is present for live cells and dead cells. A shift in current during dead-cell exposure indicates the CTx affect the glucose oxidase-based glucose sensor. The shift proved to be correctable; however, no measurable change was seen in glucose consumption due to CTx exposure.

To determine the metabolic effect of CTx on PC-12 cells, we exposed the cells to 1000 nM CTx for thirty minutes, followed by ninety minutes of recovery before treatment with alamethicin to cause the necrotic death of the cells. As a control, 1000 nM CTx was introduced to the chambers after necrotic death to determine the protein’s effect on the sensors. Figure 2a shows the raw electrochemical signals from the glucose, lactate, and oxygen sensors. A significant shift was seen in the glucose oxidase electrode signal during CTx exposure for both live and dead cells, indicating the toxin affects the glucose oxidase sensor. The shift in current at the glucose oxidase electrode during flow was quantified as the ratio of the shift from the pre-exposure value. While the magnitude of the shift in the glucose signal was less for dead cells than live cells, the ratio of the shift from the pre-exposure current was the same. These shifts indicate CTx interferes with the measurement of glucose using glucose oxidase, but as shown in Figure 2b, this small effect can be calibrated and corrected. However, after correcting for this shift, no change was seen in the rate of glucose consumption when cells were treated with CTx, even at the highest concentration and longest exposure time. For clarity, the glucose consumption rates are not shown in all following results. 

**Figure 3:**
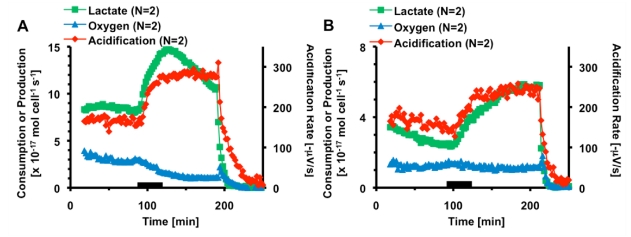
The average metabolic rates and acidification of PC-12 cells exposed to thirty minutes 1,000 nM (a) and 500 nM CTx (b). The black bar indicates the 30 minutes CTx was being flown through the cell chamber. Cell necrosis is triggered at 200 minutes.

The metabolic response of PC-12 cells exposed to 1,000 nM CTx is shown in Figure 3a. During exposure, the oxygen consumption rate steadily began decreasing, reaching 38 ± 4% of pre-exposure metabolic rates before cells were killed. The decrease in oxygen consumption was an interesting demonstration of the sensitivity of the MAMP because a previous study by Keusch and co-workers on jejunum isolated from rabbits and treated with CTx failed to show a statistically significant change in oxygen consumption [26]. When exposed to CTx, the extracellular acidification rate increased rapidly to a plateau of 169 ± 14% of the pre-exposure rate and remained stable until necrosis began. Lactate production also quickly increased, reaching a peak of 172 ± 7%. Unlike extracellular acidification, lactate production began declining, and continued declining until necrosis was triggered. 

The metabolic rates of PC-12 cells exposed to 500 nM CTx for thirty minutes are given in Figure 3b. As in the case of exposure to 1,000 nM CTx, the lactate production and extracellular acidification rates increased rapidly, with the extracellular acidification rate reaching 143 ± 11% of the pre-exposure rate within minutes of the end of exposure. During recovery, lactate production continues to increase, appearing to level off at 216 ± 11% of pre-exposure levels. Unlike 1,000 nM CTx, oxygen consumption only decreased to 75 ± 6%. The differences seen in lactate and acid production suggest that another pathway may have some effect on the metabolic profile; however, we believe the bulk of the change to be due to cAMP production. 

It is interesting to note the differences in lactate production between the 500 and 1,000 nM CTx experiments. In the 1,000 nM CTx experiment, lactate production reaches a maximum within one hour, and then begins to decline, a trend not seen in the 500 nM CTx experiment. It is possible that at sufficiently high concentrations and continuous exposure to toxin, the cell’s ability to maintain production of lactic acid is lowered. It may be that the lactate production of the PC-12 cells exposed to 500 nM CTx would have followed the same trend if a longer post-exposure time was allowed.

While studies have reported noticeable lag-times in measurable production of cAMP [7], studies of primary and immortalized neuronal cell lines have reported lag periods as short as ten minutes [27]. These studies also showed that lag-time was dependent on GM1 density, temperature, and stages in neuronal development [27,28]. The rapid increase in lactate and acid production within minutes suggests that the cells have switched to anaerobic respiration, most likely due to cAMP production *via* continuous stimulation of adenylate cyclase. In order to test the theory, an optimal dosage for use in our system was determined before inhibition experiments were performed.

**Figure 4:**
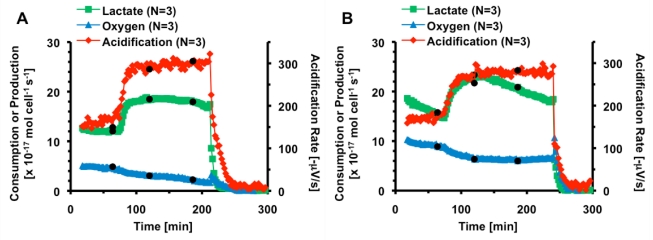
The average metabolic rates and acidification of PC-12 cells exposed to three 2-minute pulses of (a) 1,000 nM CTx (●) and (b) 100 nM CTx (●). Cell necrosis is triggered at 200 minutes.

Based on the dramatic metabolic changes seen in PC-12 cell metabolism upon a thirty minute exposure to CTx, the concentration and duration of CTx exposure were studied, as well as the effect of multiple exposures. The average metabolic responses of three chambers of PC-12 cells to three exposures of 1,000 or 100 nM CTx, with each exposure being 2 minutes long, *i.e.*, a single stop flow period, and followed by a recovery period, are given in Figure 4. 

**Figure 5:**
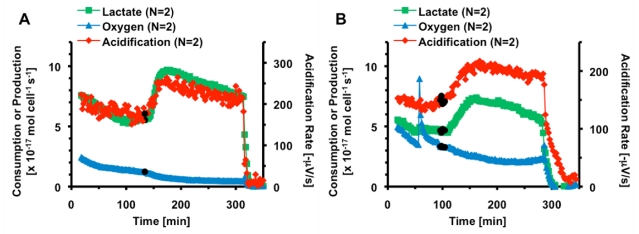
Average metabolic responses of two chambers of PC-12 cells exposed to (a) one 2 minute dose of 100 nM CTx (●) or (b) a ten minute dose of 5 nM CTx (●). The spike in oxygen at 58 minutes is the result of noise spike in one of the chambers. Cell necrosis is triggered near 300 minutes.

Increases in extracellular acidification (176 ± 14%) and lactate production rates (152 ± 3%) and decreases in oxygen consumption rates (39 ± 4) are similar to thirty minute exposure of 1,000 nM CTx, and no additional stimulatory or inhibitory effect from successive exposures is observed. When cells were exposed to 100 nM CTx for three cycles of 2 minutes each, acidification rose to 164 ± 13% and oxygen consumption decreased to 67 ± 3%. Lactate production increased to 131 ± 4% of pre-exposure rates. While it appears that lactate production is decreasing after reaching a peak, the rate of lactate production decrease prior to exposure suggests that the lactate production reaches a plateau. The same phenomenon is seen in Figure 5a. This decrease in rates can be due to equilibration of both the sensors and the cells in the MAMP, and is not typically linearized to avoid correcting for changes in cellular activity. 

In both cases, the metabolic rates do not respond to the second or third exposure; all of the change appears to come from the first exposure. In order to verify this, PC-12 cells were exposed to 100 nM CTx for a single cycle followed by a three hour recovery period. The metabolic responses are shown in Figure 5a. 

Within minutes, increases in lactate production (172 ± 4%) and acid production (148 ± 15%) were observed. The significance of this experiment was that the same metabolic response was seen whether cells were exposed to a single dose of CTx or multiple doses and no response was seen to the second or third dose of CTx in the multiple exposure experiment. This coincides with previous studies that showed that the maximal response in terms of cAMP production could be achieved with doses as low as 100 nM [29]. For this reason, our experiments were performed using 100 nM CTx as we wished to see the maximal metabolic effect using the lowest concentration and short exposure duration. PC-12 cells were also exposed to 5 nM CTx, which is in the lower portion of the dose response profile developed by Cassel [29]. 

As Figure 5b shows, when PC-12 cells were exposed to 5 nM CTx for 10 minutes, irreversible stimulation is still observed, with lactate production increasing to 153 ± 5% and acidification to 146 ± 9%. Statistical analysis showed this change to be within the range expected for higher doses, so the lower dose does not appear to lower the increase in lactate production, strengthening the argument that a continuous activation of adenylate cyclase is occurring. Unlike the higher dose experiments, no significant change in oxygen consumption was measured as a result of CTx exposure. It is possible that the change in consumption is below the sensitivity of the instrument, or only larger doses of CTx lead to mitochondrial toxicity that result in lowered oxygen consumption. 

To confirm that the metabolic response due to CTx exposure was caused by the irreversible activation of adenylate cyclase and production of cAMP, the metabolic changes caused by binding and internalization of the toxin had to be determined. To achieve this, the response of CTx was directly compared to that of CTxB, which binds to the GM1 receptors and trafficks to the ER, but does not have any inherent toxic ability. 

**Figure 6:**
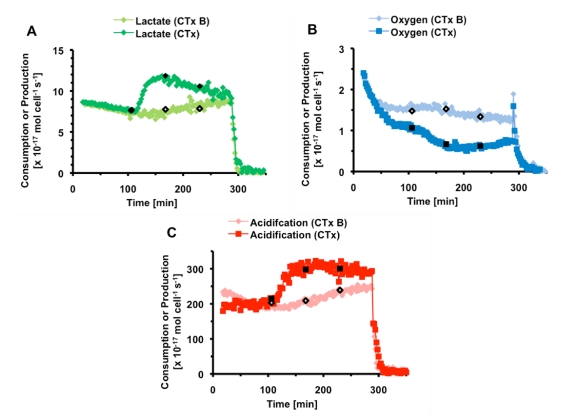
Average metabolic responses of PC-12 cells exposed to either 100 nM CTx or 500 nM CTxB subunit. Cell necrosis is triggered at 300 minutes. (a) Lactate production. The average of four chambers (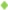) treated with three 2-minute pulse of 500 nM CTxB (◊). The other trace is the average of four chambers (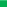) treated with three 2-minute pulse of 100 nM CTx (♦). (b) Oxygen Consumption. The average of four chambers (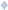) treated with three 2-minute pulse of 500 nM CTxB (◊). The other trace is the average of three chambers (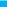) treated with three 2-minute pulse of 100 nM CTx (■). (c) Acid production. The average of four chambers (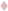) treated with three 2-minute pulse of 500 nM CTxB (◊). The other trace is the average of four chambers (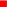) treated with three 2-minute pulse of 100 nM CTx (■).

As previously discussed metabolic rates and responses can vary slightly between passages and seedings. In order to confirm that CTx and CTxB subunit have different effects on the PC-12 cell line, the exposure paradigm of three 2-minute pulses spaced one hour apart two chambers of cells was used, with two chambers receiving 100 nM CTx and two chambers receiving 500 nM CTxB subunit; This experiment was performed twice. Figure 6 compares the average metabolic response of both CTx and CTxB subunit for lactate production (6a), and oxygen consumption (6b), and acidification (6c). For CTxB, a gradual increase in lactate to 116 ± 4% and acid production to 118 ± 11% over the combined three hour exposure and recovery period can be seen, but does not cause the initial response seen in the CTx metabolic profiles. In addition, a smaller gradual change was seen in oxygen consumption due to CTxB with a decrease to 84± 6%. The decrease of oxygen due to CTx occurred within minutes of the first pulse, with consumption reaching 53 ± 7% of basal rates at its peak, and 66 ± 6% of basal rates at the end of three hours. 

While cellular metabolism appears to be affected by the CTxB subunit, it is clear that the binding of the B subunit to the GM1 ganglioside is not the cause of the immediate changes seen when PC-12 cells are exposed to whole CTx. This supports our theory that the A subunit is reaching the cytosol and adenylate cyclase is getting activated without an appreciable lag-time. 

**Figure 7:**
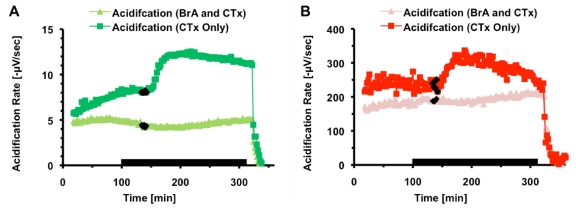
The metabolic response of PC-12 cells pre-treated with 1 µg/mL BrA. Necrosis is triggered at 330 minutes. (a) Lactate production. One chamber (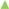) treated with brefeldin A (black bar), and then exposed to ten minutes of 10 nM CTx (♦). The lactate sensor in the replicate chamber failed to calibrate, so N = 1. The other chamber (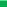) received only CTx. (b) Acid production. The average of two chambers (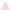) treated with brefeldin A (black bar), and then exposed to ten minutes of 10 nM CTx (♦). The other chamber (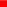) received only CTx.

After confirming that GM1 binding was not the cause of the switch to anaerobic respiration, brefeldin A (BrA) was used to disrupt vesicular transport and prevent retrograde trafficking of the toxin to the ER, thus preventing activation of adenylate cyclase and increase in cAMP in epithelial and neuronal cell lines. [18,19] In this experiment, two chambers were pre-treated with 1 µg/mL brefeldin A for thirty minutes prior to 10 nM CTx exposure. Due to the inhibitor’s reversibility, treatment continued throughout the course of the experiment, and can be seen in Figure 7. As shown, BrA successfully inhibited the increase in lactate and acid production. No significant changes were seen in oxygen consumption. The metabolic rates of all three analytes of the control chamber receiving only BrA did not change in a statistically significant manner throughout the course of the experiment (data not shown), indicating that brefeldin A does not have a measurable effect on cellular metabolism. 

Having shown that measured response were not due to binding or transport of CTx of CTxB, the role of cAMP-dependent protein kinase (PKA), was explored. PKA, involved in the regulation of glycogen and lipid metabolism [30], is activated as cAMP levels increase, and may be responsible for the metabolic effects we see in response to CTx. In order to investigate the role that PKA may play in the metabolic responses observed in response to CTx, the cells were pre-treated with H-89, which has been shown to inhibit PKA in PC-12 cells [17].

**Figure 8:**
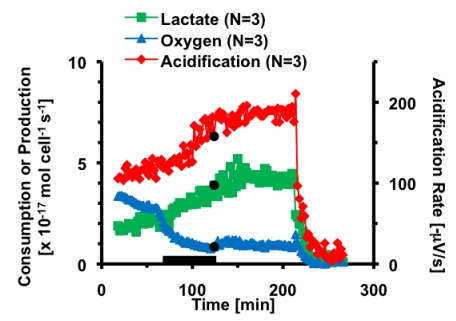
The average metabolic rates and acidification of PC-12 cells exposed to thirty minutes 30 µM H-89 (black bar) and a single two minute dose of 100 nM CTx (♦). Cell necrosis is triggered at 220 minutes.

The metabolic response of PC-12 cells treated with 30 µM H-89 for 1 hour followed by a single 2 minute exposure to 100 nM CTx is shown in Figure 8. During treatment with H-89, there is a gradual increase in anaerobic respiration. By the end of the thirty minute exposure, but prior to CTx exposure, lactate production had increased to 166 ± 24% of pre-treatment rates and extracellular acidification rates reached 167 ± 22%. H-89 appears to have successfully inhibited further lactate and acid increase, as no statistically significant changes occurred after CTx exposure. During H-89 exposure, the oxygen consumption rate decreased to 50% of pre-exposure rates. Upon treatment with 100 nM CTx, the rate continued to decrease to 33 ±3% of pre-inhibitory rate. This decrease is within the range expected for 100 nM CTx, however the results seem to indicate that this decrease was due to H-89 exposure. This suggests that the increase in lactate and acid production is mediated by PKA. However, H-89 also inhibits a number of additional protein kinases to 25% or less of their normal activity including MAPKAP-K1b, MSK1, PKBα, SGK, S6K1, ROCK-II, AMPK, and CHK1 [31,32]. For this reason, a more specific inhibitor of PKA could be used to prove the link between the metabolic signals seen and increased phosphorylation of glycogen, but these specific inhibitors are difficult to find. 

**Figure 9:**
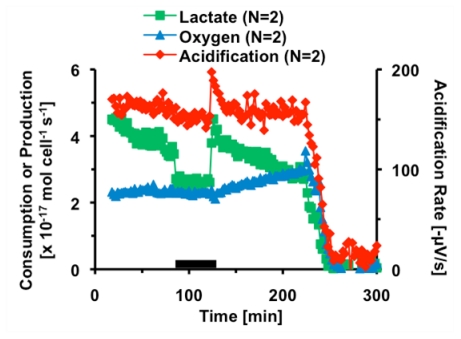
The average metabolic response of PC-12 cells exposed to thirty minutes 10 µM forskolin. The black bar indicates the 10 µM forskolin was being flown through the cell chamber. Cell necrosis is triggered at 230 minutes.

In order to further investigate the neuronal response to CTx, the metabolic response of forskolin, a direct and reversible activator of adenylate cyclase, was determined and the metabolic profiles compared. PC-12 cells were treated with 10 µM forskolin for 30 minutes; the metabolic response is shown in Figure 9 and can be compared to the 30 minute exposure of CTx shown in Figure 3a. The response of PC-12 cells to forskolin was quite different than the response to CTx. During exposure to and recovery from forskolin, there is no significant change in the extracellular acidification rate of the cells. Lactate production decreases to 69 ± 7% pre-exposure activity during exposure to forskolin. After forskolin is removed, the lactate production increases briefly to 92 ± 8% of pre-exposure levels before returning to the basal level. Based on the decreasing lactate signal seen in the pre-exposure period, it appears that lactate production returned to basal levels after forskolin was removed, like acid production did. There is no significant change in the consumption of oxygen during exposure, but once forskolin is removed, oxygen consumption steadily increases, reaching 125 ± 4% of pre-exposure levels after two hours. These differences compared to CTx may be explained by the fact that forskolin has secondary activities in addition to its effects on adenylate cyclase. Forskolin has been shown to interact with glucose transporters and ion channels [33], which could potentially lead to additional metabolic effects beyond those caused by elevated cAMP. In conclusion, it is clear that while many pharmacological and toxicological studies rely on the similarities of increased cAMP production using CTx and forskolin, the metabolic pathways affected by each stimulant are significantly different, and care should be used in the analysis of the resulting metabolism and cell physiology.

## 4. Conclusions

Multianalyte microphysiometry was successfully used to determine the metabolic response of PC-12 cells to CTx. When cells were treated with CTx, changes in cellular metabolism were seen in as little as ten minutes as increases of extracellular acidification and lactate production, and decreases in oxygen consumption. Using the MAMP, we were able to use inhibitors to demonstrate that cAMP production is a likely cause for this effect. The lack of immediate response of cells to the B subunit of CTx showed that binding of toxin to GM1 receptors was not the cause of the large changes in metabolism. Disruption of vesicular transport with brefeldin A showed that transport of the toxin to the ER is required for the metabolic response. These experiments together show that the metabolic response seen occurs only after the toxin has been transported. H-89, a PKA inhibitor, was successfully able to inhibit the metabolic response of CTx, indicating that this cAMP-dependent kinase was involved in the increase in anaerobic respiration as measured by the MAMP. Interestingly, the metabolic response of PC-12 cells to forskolin did not mirror the response to CTx. These experiments show that the MAMP is a useful tool for determining the acute metabolic effects of biological toxins in real-time and for exploring the signaling pathways triggered by exposure to a toxin or other agent

Future work in the study of the metabolic effects of CTx using the MAMP will center on the use of the Caco-2 or T84 epithelial colon cell lines to determine whether there is a differential metabolic response to CTx between the colon cells and the enteric nerve cells that have been implicated in up to 50% of the clinical effects of cholera [8,20]. It may also be possible to incorporate a Cl^-^ sensor into the MAMP sensor head, which would allow us to track chloride secretion as the acid and lactate production increase, which would help further confirm that the metabolic response seen is due to cAMP production.
